# Environmental exposures and chronic inflammatory dermatoses: Preventive and therapeutic strategies

**DOI:** 10.1111/srt.70009

**Published:** 2024-08-12

**Authors:** Isabella J. Tan, Aarushi K. Parikh, Bernard A. Cohen

**Affiliations:** ^1^ Rutgers Robert Wood Johnson Medical School New Brunswick New Jersey USA; ^2^ Department of Dermatology The Johns Hopkins Hospital Baltimore Maryland USA

**Keywords:** allergens, atopic dermatitis, climate, environment, inflammation, therapeutic

## 1 BACKGROUND

Chronic inflammatory dermatoses, including contact dermatitis and eczema, affect approximately 20% of the population and impose significant burdens on patients.^1^ These conditions are aggravated by environmental factors such as allergens, irritants, pollutants, and climatic conditions.^2^ Recent research highlights the critical role of these exposures in disease pathogenesis, leading to advancements in preventive and therapeutic strategies. Despite progress, challenges remain, especially in providing equitable access to optimal treatments for underserved populations. Long‐term effects of environmental exposures, including persistent inflammation and increased disease progression, underscore the necessity for continued research to elucidate the underlying molecular mechanisms. This manuscript aims to examine preventive and therapeutic strategies, evaluate epidemiological data and prevalence, and discuss the significant impact of these conditions on patients' quality of life, emphasizing the need for improved patient care and reduction of the overall burden of chronic inflammatory dermatoses.

## 2 METHODS

Articles were identified through a PubMed search conducted using the search algorithm: (“environment” OR “pollution” OR “allergen” OR “climate”) AND (“acne” OR “eczema” OR “dermatitis”). Clinical studies and commentaries were included, while secondary sources such as reviews were consulted to supplement potential gaps in information. Articles were considered eligible if they addressed the specified environmental factors in relation to acne, eczema, or dermatitis.

## 3 ENVIRONMENTAL EXPOSURES AND CHRONIC INFLAMMATORY DERMATOSES

**FIGURE 1:**
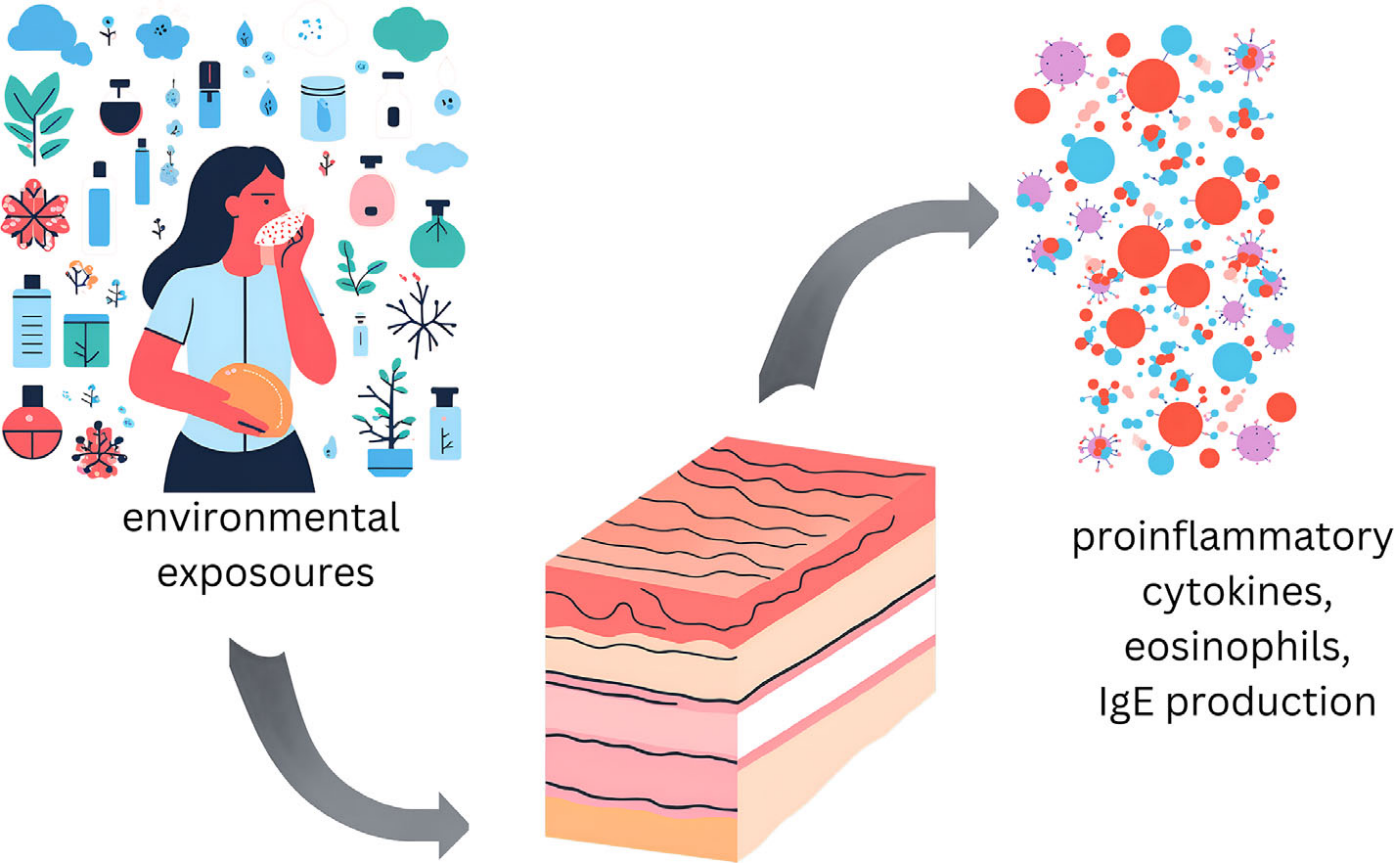
Pathogenesis of atopic dermatitis exacerbation with allergens.

Allergens, such as nickel and fragrances found in jewelry and personal care products, significantly contribute to inflammatory dermatoses like atopic dermatitis and allergic contact dermatitis by triggering immune responses mediated by T‐cells and cytokines (Table 1).^3^ Clinical management involves allergen identification, avoidance, and topical treatments, with severe cases potentially requiring systemic therapies (Figure 1). Irritants in soaps, detergents, solvents, and chemicals disrupt the skin barrier, causing irritant contact dermatitis or exacerbating eczema and occupational dermatoses; management includes avoidance, emollients and, in severe cases, corticosteroids.^4^ Environmental pollutants, including particulate matter <2.5 µm, may aggravate skin conditions such as acne and psoriasis by 34% to 121% due to oxidative stress and inflammation.^5^ Indoor pollutants like fungal spores and outdoor pollutants like hydrocarbons also worsen these conditions. Climatic factors, including extreme temperatures, further exacerbate dermatoses by affecting skin moisture and microbial growth.^6^

**TABLE 1:** Common associations between environmental exposures and chronic inflammatory dermatoses and their associated preventive strategies and therapeutic interventions.

Environmental exposure	Associated inflammatory dermatosis	Prevention and therapeutic strategies
Allergens	Atopic dermatitis, allergic contact dermatitis	Identify and avoid allergens (e.g., nickel, fragrances). Use hypoallergenic and fragrance‐free products. Educate on allergen sources and symptoms.
Irritants	Irritant contact dermatitis, acne, occupational dermatoses	Avoid irritants (e.g., soaps, detergents, solvents). Use protective gear (gloves, masks). Choose gentle, non‐irritating products. Use emollients and moisturizers. Consider corticosteroids for severe cases. Follow public health measures for practical avoidance strategies
Indoor pollutants	Atopic dermatitis, contact dermatitis, acne	Reduce indoor allergens and pollutants (e.g., fungal spores). Use air purifiers. Maintain clean, mold‐free environments. Improve indoor air quality through proper ventilation. Apply moisturizers to protect the skin barrier.
Outdoor pollutants	Atopic dermatitis, contact dermatitis, acne	Limit outdoor exposure during high pollution periods. Use “anti‐pollution” skincare products with antioxidants. Shower and change clothes after exposure. Use wearable sensors for real‐time environmental monitoring.
Climate	Atopic dermatitis, acne, psoriasis, rosacea	Adapt skincare routines based on climate (e.g., heavier moisturizers in cold weather, lighter products in humid weather). Protect skin from extreme temperatures. Use AI and genomics for personalized skincare solutions based on climate.

## 4 PREVENTIVE AND THERAPEUTIC STRATEGIES

### 4.1 Prevention

**FIGURE 2:**
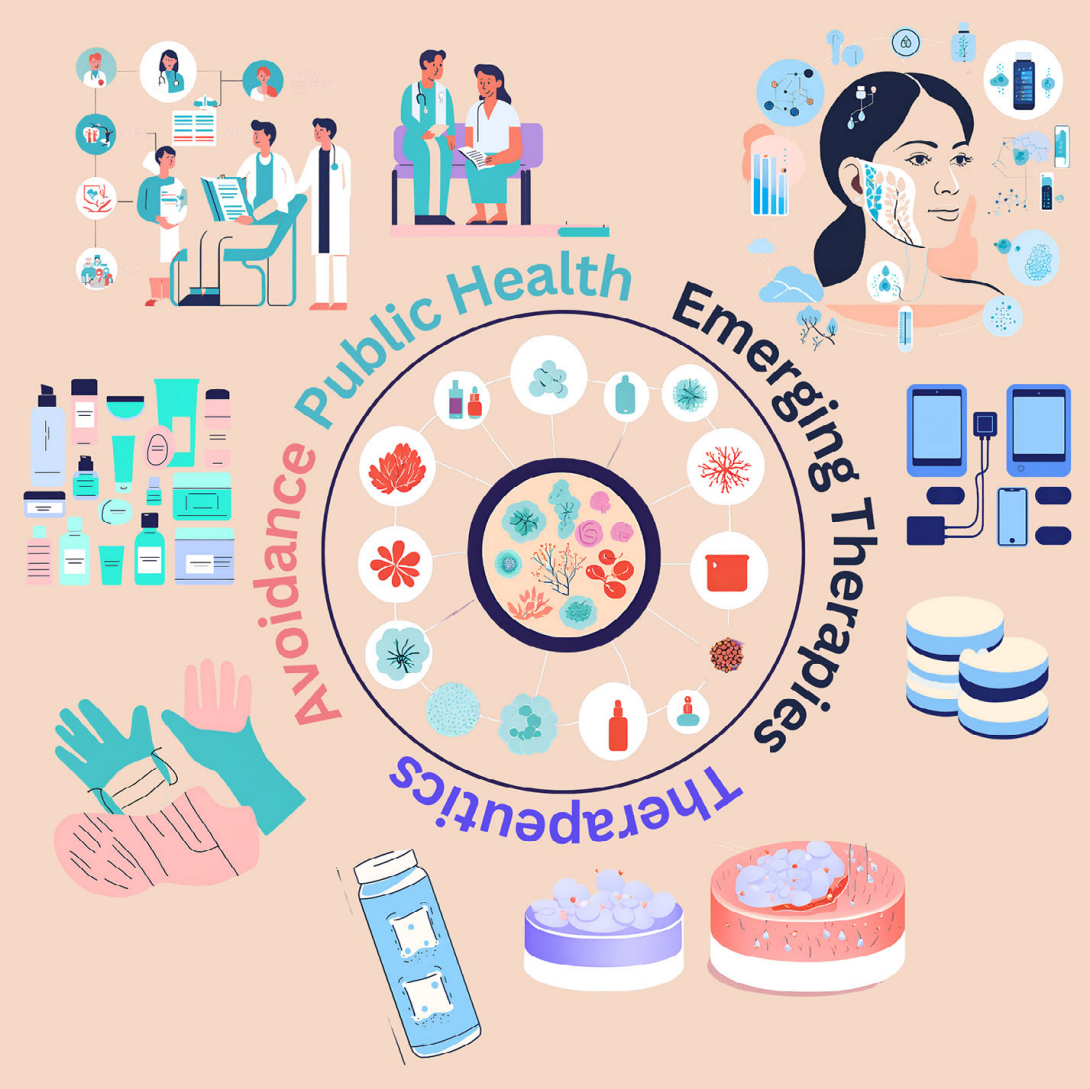
Preventive and therapeutic strategies for mitigating environmental exposures and inflammatory dermatoses.

Effective prevention strategies rely on targeted public health measures and comprehensive patient education (Figure 2). Recent public health initiatives emphasize educating individuals about prevalent allergens and irritants through channels like community workshops, online resources, and healthcare provider interactions. These efforts aim to enhance awareness and empower individuals to identify and minimize exposure to potential triggers in their everyday household products, personal care items, and occupational settings.

#### 4.1.1 Public health measures and education

Public health campaigns focus on raising awareness about common allergens such as nickel and fragrances for patients at risk due to primary inflammatory dermatoses. Educational materials highlight specific avoidance strategies, encouraging individuals to read ingredient labels meticulously and opt for hypoallergenic and fragrance‐free alternatives when possible. Health professionals play a pivotal role in providing personalized advice on allergen avoidance and recommending suitable skincare routines to mitigate dermatoses risk.

#### 4.1.2 Avoidance strategies

Recent public health initiatives recommend practical avoidance strategies to limit exposure to allergens and irritants. These strategies include using personal protective equipment (e.g., gloves, masks) during exposure‐prone activities, adopting gentle cleansers and moisturizers to maintain skin barrier function, and using “anti‐pollution” skincare products with antioxidants like vitamin C, vitamin E, and niacinamide.^7^ Additionally, the National Eczema Association offers a dedicated Eczema Management webpage and fact sheets for improved management.

Emerging approaches also suggest incorporating smart technology and personalized data into skin care regimes. Wearable sensors, for example, can monitor environmental exposure in real‐time, alerting users to high levels of pollutants in the air for timely protective measures. Personalized skincare solutions powered by artificial intelligence and genomics can analyze individual skin types and environmental sensitivities to recommend custom formulations.

### 4.2 Therapeutic interventions

Therapeutic interventions encompass a range of pharmacological and non‐pharmacological approaches to effectively manage dermatoses. Pharmacologically, topical corticosteroids serve as the cornerstone for reducing inflammation.^8^ Emollients and moisturizers restore the skin barrier function and prevent moisture loss. Calcineurin inhibitors provide relief from inflammation in areas with sensitive skin, such as the eyelids, face, groin, and armpits, where the skin is delicate and prone to irritation.^8^ Non‐pharmacological approaches—as wet wraps over topical treatments that enhance hydration and medication absorption; barrier creams to shield skin from irritants—complement pharmacological therapies.^8^

### 4.3 Challenges and considerations

Many of these environmental exposures are more common in specific occupations and urban areas. Effective treatment not only involves personalization to the patient's circumstances, but also mitigating disparities in healthcare access and economic constraints. High costs for prescription medications, dermatological consultations, and specialized skincare create a significant financial burden for individuals with chronic dermatological conditions. One study identified that individuals with atopic dermatitis report a median annual out‐of‐pocket expense of $600, with 41.9% spending $1000 or more, which included costs for healthcare provider visits (68.7%), prescription co‐pays (64.3%), and nonprescription products like moisturizers (94.3%).^9^ Implementing preventive measures may also function to reduce the need for prescription and non‐prescription medications thus, limiting healthcare costs.

Addressing access inequities demands targeted interventions, such as policy initiatives to improve healthcare accessibility and affordability, and community outreach to educate and support at‐risk populations. The cumulative impact of chronic exposure to allergens, irritants, pollutants, and climatic conditions on skin health is not fully understood, particularly regarding how these interactions affect skin health chronically and which biomarkers and molecular mechanisms are involved, underscoring the need for further research and longitudinal studies.

## 5 CONCLUSION

This research highlights the significant role of targeted preventive and therapeutic strategies for environmental factors in managing chronic inflammatory dermatoses like contact dermatitis and eczema. Effective management involves public health education, avoidance strategies, and a combination of pharmacological and non‐pharmacological approaches. Further research is needed to clarify molecular mechanisms of these triggers and develop targeted therapies. Clinicians should incorporate environmental exposure management into care, and public health efforts must focus on improving awareness and access to treatments to alleviate the burden of these conditions.

## CONFLICT OF INTEREST STATEMENT

The authors declare no conflicts of interest.

## IRB APPROVAL STATUS

Not applicable.

## REPRINT REQUESTS

No reprints requested.

## PATIENT CONSENT

Not applicable.

## Data Availability

The data that support the findings of this study are found through the PubMed algorithm supplied in the manuscript. These data were derived from the following resources available in the public domain: https://pubmed.ncbi.nlm.nih.gov/.
